# Modified Bidirectional Encoder Representations From Transformers Extractive Summarization Model for Hospital Information Systems Based on Character-Level Tokens (AlphaBERT): Development and Performance Evaluation

**DOI:** 10.2196/17787

**Published:** 2020-04-29

**Authors:** Yen-Pin Chen, Yi-Ying Chen, Jr-Jiun Lin, Chien-Hua Huang, Feipei Lai

**Affiliations:** 1 Graduate Institute of Biomedical Electronics and Bioinformatics National Taiwan University Taipei City Taiwan; 2 Department of Emergency Medicine National Taiwan University Hospital Chu-Tung Branch Hsinchu County Taiwan; 3 Department of Emergency Medicine National Taiwan University Hospital Taipei City Taiwan; 4 Department of Emergency Medicine College of Medicine National Taiwan University Taipei City Taiwan; 5 Department of Computer Science & Information Engineering National Taiwan University Taipei City Taiwan; 6 Department of Electrical Engineering National Taiwan University Taipei City Taiwan

**Keywords:** transformer, BERT, deep learning, emergency medicine, automatic summarization

## Abstract

**Background:**

Doctors must care for many patients simultaneously, and it is time-consuming to find and examine all patients’ medical histories. Discharge diagnoses provide hospital staff with sufficient information to enable handling multiple patients; however, the excessive amount of words in the diagnostic sentences poses problems. Deep learning may be an effective solution to overcome this problem, but the use of such a heavy model may also add another obstacle to systems with limited computing resources.

**Objective:**

We aimed to build a diagnoses-extractive summarization model for hospital information systems and provide a service that can be operated even with limited computing resources.

**Methods:**

We used a Bidirectional Encoder Representations from Transformers (BERT)-based structure with a two-stage training method based on 258,050 discharge diagnoses obtained from the National Taiwan University Hospital Integrated Medical Database, and the highlighted extractive summaries written by experienced doctors were labeled. The model size was reduced using a character-level token, the number of parameters was decreased from 108,523,714 to 963,496, and the model was pretrained using random mask characters in the discharge diagnoses and International Statistical Classification of Diseases and Related Health Problems sets. We then fine-tuned the model using summary labels and cleaned up the prediction results by averaging all probabilities for entire words to prevent character level–induced fragment words. Model performance was evaluated against existing models BERT, BioBERT, and Long Short-Term Memory (LSTM) using the Recall-Oriented Understudy for Gisting Evaluation (ROUGE) L score, and a questionnaire website was built to collect feedback from more doctors for each summary proposal.

**Results:**

The area under the receiver operating characteristic curve values of the summary proposals were 0.928, 0.941, 0.899, and 0.947 for BERT, BioBERT, LSTM, and the proposed model (AlphaBERT), respectively. The ROUGE-L scores were 0.697, 0.711, 0.648, and 0.693 for BERT, BioBERT, LSTM, and AlphaBERT, respectively. The mean (SD) critique scores from doctors were 2.232 (0.832), 2.134 (0.877), 2.207 (0.844), 1.927 (0.910), and 2.126 (0.874) for reference-by-doctor labels, BERT, BioBERT, LSTM, and AlphaBERT, respectively. Based on the paired t test, there was a statistically significant difference in LSTM compared to the reference (*P*<.001), BERT (*P*=.001), BioBERT (*P*<.001), and AlphaBERT (*P*=.002), but not in the other models.

**Conclusions:**

Use of character-level tokens in a BERT model can greatly decrease the model size without significantly reducing performance for diagnoses summarization. A well-developed deep-learning model will enhance doctors’ abilities to manage patients and promote medical studies by providing the capability to use extensive unstructured free-text notes.

## Introduction

### Background

Medical centers are the last line of defense for public health and are responsible for educating medical talent. The number of patients in the emergency department of such medical centers is particularly large, and these patients tend to have more severe conditions than those admitted to hospital at a lower tier. For staff, the emergency department can be an overloaded work environment [1,2]. At the beginning of the shift, a doctor must perform primary care for more than 30 patients who remain in the emergency department from less than 1 hour to more than 3 days, while simultaneously treating new arrivals from triage. The conditions of patients in the emergency department also tend to change rapidly, and the staff must be able to handle these patients under time constraints. The International Statistical Classification of Diseases and Related Health Problems (ICD) codes [3] and recent discharge diagnoses can help staff rapidly determine baseline conditions. However, in a medical center, patients may have multiple underlying diseases and several comorbidities that were previously recorded as ICD codes and discharge diagnoses in electronic health records (EHRs). Because ICD codes only reflect the disease and not the associated treatments, this lack of information limits the ability of medical staff to consider information related to a previous hospital visit. Occasionally, ICD codes are selected imprecisely and do not adequately represent the condition of the patient. Therefore, discharge diagnoses are required for staff to become familiar with a patient’s condition. However, the number of words describing these details in a diagnostic sentence can vary widely. Consequently, the attending physician in the emergency department may have to read as many as 1500 words to cover the medical history of all patients under their charge. To resolve this challenge, the purpose of this study was to establish a diagnostic summary system to help hospital staff members check information on all patients more quickly.

### Related Works

There are several available methods to accomplish a text summarization task, ranging from traditional natural language processing (NLP) to deep-learning language models [4-9]. The goals of previous text summarization studies in the medical field [5] included finding information related to patient care in the medical literature [5,10-13], identifying drug information [14], determining medical article topic classifications [15], and summarizing medical articles [16]. In the majority of cases, data sources for the automatic summarization task were medical articles [16] such as PubMed articles [5,11,14,15]. In recent years, EHRs have been widely adopted in several hospitals and clinics, and additional data sources such as the Medical Information Mart for Intensive Care III [17] dataset are available online for free and promote medical progress. Based on medical record research, the monitoring of several disease indicators, clinical trial recruitments, and clinical decision making, several clinical summarization systems based on EHRs have been studied [4,18-20]. However, no studies have addressed the issue of a diagnostic summary system to help hospital staff access information on all patients in their care more quickly.

Although EHRs provide useful information, the majority of this information is recorded as free text, making it challenging to analyze along with other structured data [4]. In recent years, NLP and deep-learning approaches have flourished, furnishing health care providers with a new field to promote human health. Several excellent language models are now available to help machines analyze free text. One such model is Bidirectional Encoder Representations from Transformers (BERT) [21], which is an extension of Transformer [22], and received the highest score for several NLP tasks [21,23,24].

Transformer is a state-of-the-art model, which was released to translate and improve the efficiency of Long Short-Term Memory (LSTM) [25]-based language models [22]. Similar to many deep-network models, Transformer has an encoder and a decoder. The encoder converts the input data into meaningful codes (vector or matrix), while reducing the dimension size (a major bottleneck for data analysis), and the decoder converts the code to output [26]. Taking translation as an example, the encoder converts an English sentence into a digital vector in latent space, and the decoder then converts the digital vector into a corresponding sentence in the desired language. The encoder of Transformer has an embedding model, a repeating block model with a multihead self-attention model, and a feedforward model with an architecture based on the shortcut connections concept [27] and layer normalization [22,28].

The automatic text summarization task has two branches: extractive and abstractive [29]. The extractive branch identifies keywords or sentences as summaries without changing the original document, while the abstractive branch adapts a new short sentence. The diagnosis summarizes the entire admission course, including the chief complaints and treatment course, in highly concentrated and meaningful sentences that help other staff members to quickly manage patients. Because patients in the emergency department have many underlying diseases, along with the high complexity of the conditions of individual patients, incomplete sentences, grammatical issues, and some subordinate prompts, the diagnosis obtained may not be concise. Consequently, the staff needs to include an abundance of words in their diagnoses to best represent the condition of the patient. These rich vocabularies involve not only specific disease terms but also important treatments that are delivered in the course of admission and are associated with verbose text related to diagnoses. Therefore, it is necessary to further summarize the diagnoses using an extractive summarization approach.

The extractive summarization model can be simplified to a regression problem that outputs the probability of choosing or not choosing. Taking a single character as the token unit, this problem is similar to the segmentation problem in computer vision [30,31], which outputs the class probability by pixels. A BERT-based model is the superior choice in this context since the attention weight is similar to the extraction probability [32,33] and Transformer was reported to exhibit higher performance with the language model than convolutional neural networks, recurrent neural networks, or the LSTM model [22].

BERT is a state-of-the-art language model for many NLP tasks that is pretrained with unsupervised learning, including “masked language modeling” and “next-sentence prediction.” BERT is pretrained through several corpus datasets, which are then transferred to learning through supervised data [34,35] to defeat other language models in several competitions [21,36]. The pretrained model is available [37] and can be fine-tuned for many scenarios.

Because English is not the native language in Taiwan, there are various typos and spelling errors in free-text medical records. Use of the word-level method [38], which is based on Word2vec [39,40], can result in this out-of-vocabulary obstacle. In addition, the internal structure of the word is also important and improves vector representation [41,42]. This obstacle can be overcome by adopting the character-level method [40,43,44], which uses a single character or letter as the analysis unit, or the byte-pair encoding (BPE) model, which breaks down each word into multiple subword units (ie, “word pieces”) [45]. These methods can decrease the total vocabulary and can also handle rare words, typos, and spelling errors. The word-level and BPE methods were adopted in BERT, resulting in a comprehensive and adaptable model for many types of NLP tasks.

In EHRs, medical terms, abbreviations, dates, and some count numbers for treatment are rarely found in the general corpus dataset, and will result in poor performance of the model. BioBERT, which is based on the BERT model and uses the same tokenizer, is obtained through advanced training on a biomedical corpus [46], and was considered to be well-suited to address our study aims. However, the general computing environments of some medical centers have limited capability to train or fine-tune a heavy model (involving approximately 1 billion parameters) in BERT. Therefore, replacing token units with a character-level method can further reduce the vocabulary and model size, enabling the use of the internal structures of words to avoid the out-of-vocabulary problem.

### Objective

Our goal was to build a diagnoses-extractive summarization model that can run on the limited computing resources of hospital information systems with good performance. Therefore, we present AlphaBERT, a BERT-based model using the English alphabet (character-level) as the token unit. We compared the performance of AlphaBERT and the number of parameters with those of the other existing models described above.

## Methods

### Materials

A dataset of 258,050 discharge diagnoses was obtained from the National Taiwan University Hospital Integrated Medical Database (NTUH-iMD). The discharge diagnoses originated from the following departments (in descending order): surgery, internal medicine, obstetrics and gynecology, pediatrics, oncology, orthopedic surgery, urology, otolaryngology, ophthalmology, traumatology, dentistry, neurology, family medicine, psychiatry, physical medicine and rehabilitation, dermatology, emergency medicine, geriatrics, and gerontology. This study was approved by Research Ethics Committee B, National Taiwan University Hospital (201710066RINB).

**Figure 1:**
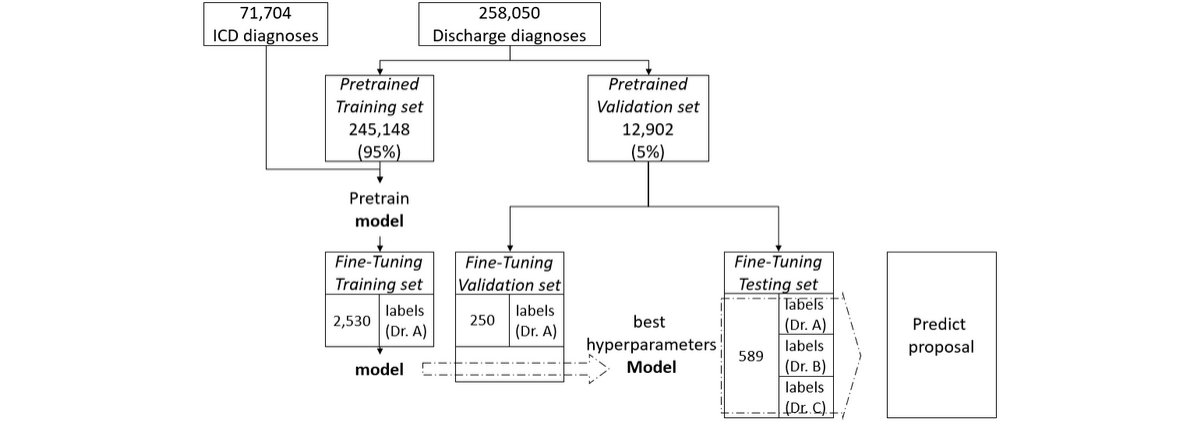
Pretrained validation dataset. ICD: International Statistical Classification of Diseases and Related Health Problems.

In the pretraining stage, 71,704 diagnoses collected by the ICD 10th Revision (ICD-10) [3] were also used, and the 258,050 discharge diagnoses were split into 245,148 (95.00%) as the pretrained training dataset and 12,902 (5.00%) as the pretrained validation dataset. In the fine-tuning stage, the extractive summary for supervised learning was labeled by three experienced doctors who have worked in the emergency department for more than 8 years. The fine-tuned dataset included 2530 training labels from the pretrained training dataset, and 250 validation labels and 589 testing labels from the pretrained validation dataset (Figure 1). We fed the model using 589 data entries in the fine-tuning testing set and obtained a predicted proposal for performance evaluation.

### Implementation Details

The hardware used for implementation was an I7 5960x CPU, with 60 G RAM, and 2 Nvidia GTX 1080 Ti GPUs. The software used were Ubuntu 18.04 [47], Anaconda 2019.03 [48], and PyTorch 1.2.0 [49].

### Label Data

We created a diagnosis-label tool to print the discharge diagnosis from the dataset in a textbox. Doctors highlighted the discharge diagnoses by selecting words that were considered to be most relevant, and the tool identified the highlighted position characters, which were labeled 1 and the others were labeled 0. For example, “1.Bladder cancer with” was labeled “001111111111111110000” and stored in the label dataset. We encouraged doctors to skip short diagnoses, because the summarization service will be more useful for longer diagnoses. Therefore, only longer diagnoses were labeled and collected in the fine-tuning set. 

### Data Augmentation

In this study, the pretraining dataset was smaller than the dataset used in the pretrained model of BERT and its extensions [21,46]. Because the diagnoses included several independent diagnoses such as hypertension, cellulitis, and colon cancer, we augmented the pretraining dataset by stitching many diagnoses derived from ICD codes or NTUH-iMD. Accordingly, data augmentation was performed by selecting between 1 and 29 random diagnostic data entries from the dataset and combining them into longer and more complex diagnoses as the pretrained dataset. We set all diagnoses to a maximum of 1350 characters because of GPU memory limitations.

Because there was also a significant shortage of fine-tuning data, the same data augmentation strategy was used to extend the fine-tuning dataset. To provide greater tolerance for typos, we also randomly replaced 0.1% of the characters in the diagnoses during the fine-tuning stage.

### Preprocess and Tokenization

We retained only 100 symbols, including letters, numbers, and some punctuation. All free-text diagnoses were preprocessed by filters, and symbols outside of the reserved list were replaced with spaces. Original letter cases (uppercase and lowercase) were retained for analysis.

The preprocessing of diagnoses then converted the symbols (letters, numbers, and punctuation) into numbers with a one-to-one correspondence. For example, “1.Bladder cancer with” was converted to the array “14, 11, 31, 68, 57, 60, 60, 61, 74, 0, 59, 57, 70, 59, 61, 74, 0, 79, 65, 76, 64.”

### Model Architecture

**Figure 2:**
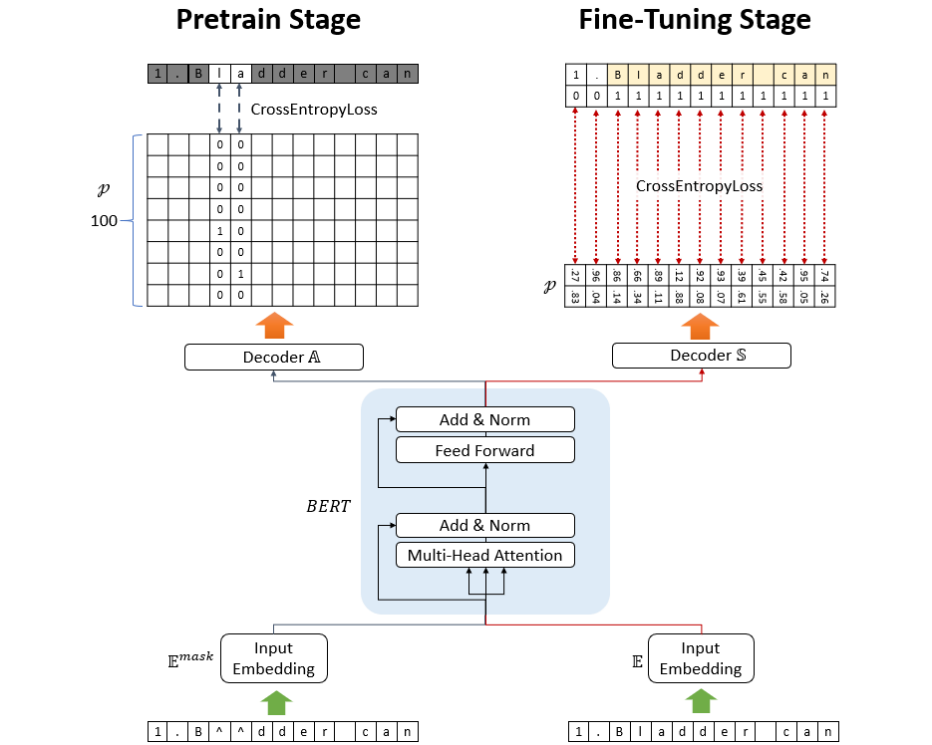
Deep-learning model architecture.

The architecture of AlphaBERT is based on that of BERT, and our model is based on the PyTorch adaptation released by the HuggingFace team [37] . In this study, we used a 16-layer Transformer encoder with 16 self-attention heads and a hidden size of 64. Character-level tokenizers were used as the token generator of AlphaBERT. There are 963,496 parameters in the whole model, and the symbols are represented by tokenization as one-hot encoding, corresponding to each vector with a hidden size of 64 as the token embeddings. The position embeddings (hidden size 64) are trainable vectors that correspond to the position of the symbol [21], in which the maximum length of position embeddings is set to 1350. The summation of the token embeddings and position embeddings is then used as the input embeddings (Multimedia Appendix 1) as input to AlphaBERT (Figure 2).

### Pretraining Stage

The two-stage learning approach of BERT [21] is based on an unsupervised feature-based method, which then transfers the learning to supervised data. The unsupervised pretraining stage of BERT uses a masked language model procedure called a “cloze procedure” [21,50]. Since AlphaBERT was used as the character-level token model, and we used “^” as the “[MASK]” in BERT, we randomly selected 15% of the character sequence, 80% of which was replaced by “^,” 10% was replaced with letters, and the remaining 10% was left unchanged. After the loss converged, we then masked the entire word to further pretrain our model.

Because the free-text diagnoses contained dates, chemotherapy cycles, cancer staging index, and punctuation marks, these words were nonprompted, nongeneric, and changed sequentially. Even experienced doctors cannot recover hidden dates or cycles without prompts, and therefore the letters were replaced with other letters, numbers were replaced with other numbers, and punctuation marks were replaced with other punctuation marks (but were still randomly selected to mask by “^”).

In the masked language model used in this study, the BERT model was connected to a fully connected network decoder **A**, which then transformed the 64-dimensional hidden size to a 100-dimensional symbol list size corresponding to the probability *p* of each symbol. The loss function *Loss^mask^* is the cross-entropy among the probabilities of each symbol (left side of Figure 2).


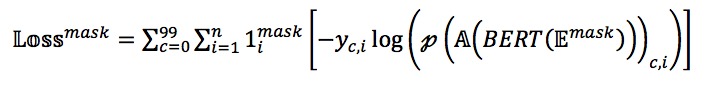


where *E^mask^* denotes the input embedding converted from masking characters, *BERT ()* is the BERT model, *A* () is the fully connected linear decoder to each preserved character, *p* is the probability function, and *1_i_^mask^* denotes the *i_th_* character masked.

### Fine-Tuning Stage

Another fully connected network, ***S****,* decoded the results of the multi-layer Transformer encoder to the predicted probability *p*. The output size of the decoder ***S*** is two-dimensional, which indicated the possibility of selection. The loss function ***Loss*** is the cross-entropy among *p* and the ground truth (right side of Figure 2).


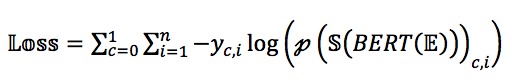


where *S* () is the full connected linear decoder for selection.

### Cleanup Method

When we evaluated our model, the probability of each word was represented by the mean probability of each character in the word. In this method, we split the characters list *C =* [*c_1_, c_2_,...c_n_*] into a list of several word sets *W* = [*w_1_, w_2_, ..., w_k_*], *k* ≤ *n*, where the cleanup probability *p̂_i_* of each *c_i_* will be the average of all probabilities in *w_m_* that contain *c_i_*.


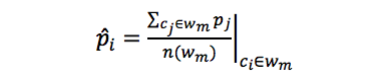


where *p* denotes the probability after clean up, *w_m_* denotes the sequences of characters belonging to the *m_th_* word, and *n*() is the length of the unit in the set.

### BERT Models for Extractive Summarization

We also compared the state-of-the-art models and adjusted them to fit the target task. The purpose of these models was not summarization, and there is no well-presented, fine-tuned model for this purpose available. Based on the word pieces BPE method [45], all words were split into several element tokens and then the predicted result was associated with the word pieces. Accordingly, for this task, we filtered out the punctuation marks and added “[CLS]” in the head of every word (*E^head^*) o represent the entire word, which prevented fragmented results.


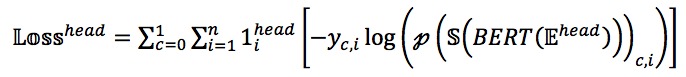


Where *E^head^* denotes the input embedding converted from a word (with head) and *1_i_^head^* denotes that the *i_th_* character is a head token.

### LSTM Model for Extractive Summarization

We also used the LSTM model [23,25] for this summarization task. To achieve effective comparison with our model, we pretrained the input embedding using Word2vec [39] and adopted a 9-layer bidirectional LSTM with 899,841 parameters, which was very similar to our model.


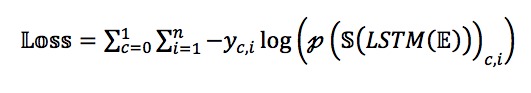


### Hyperparameters

We used Adam optimization [51] with a learning rate of 1×10^–5^ in the warmup phase [27,52,53], and then switched to a rate of 1×10^–4^ and a minibatch size of 2. The hyperparameter used in this study was the threshold to the character-level probability of selection, which was chosen using a receiver operating characteristic (ROC) curve and *F1* statistic counting from the fine-tuning validation set (Multimedia Appendix 2).

### Measurement

We measured the performance of the various models using the ROC curve, an *F1* statistic, and the *F1* statistic of Recall-Oriented Understudy for Gisting Evaluation (ROUGE) [54]. To maintain measurement consistency, we filtered out all punctuation in the predicted proposals, counted the results at the word level, and collected physicians’ feedback for each model. A questionnaire website was established in which the original diagnoses were randomly selected and displayed in the first part, and the ground truth summary proposal determined by testing labels and proposals predicted by models were displayed in the second part under random sorting. We recruited 14 experienced physicians for this purpose, including the chief resident, 10 attending physicians of the emergency department at the medical center, one emergency department attending physician at the regional hospital, and two emergency attending physicians at the district hospital. They entered a score of 0-3 for each proposal, in which 0 represented “nonsensical” and 3 represented “good.”

### Statistical Analysis

Data were analyzed using the statistical package RStudio (version 1.2.5019) based on R (version 3.6.1; R Foundation for Statistical Computing, Vienna, Austria). For group comparisons, we performed the pairwise paired *t* test on the dependent variables of the physician scores and set the significance threshold level to *P*<.05. 

## Results

The discharge diagnoses dataset included 57,960 lowercase English words. The maximum number of words in a diagnosis was 654 (3654 characters), with a mean of 55 (SD 51) words corresponding to 355 (SD 318) characters. In the fine-tuning dataset, the mean number of words in the diagnoses and summary were 78 (SD 56) and 12 (SD 7), respectively. The retention ratio [55] (ie, words in the summary divided by words in the diagnoses) was 12 out of 78 words (15%). The fine-tuning testing set included 138 diagnoses with incorrect words, and a total of 183 incorrect words were counted manually by two attending physicians, including 153 misspellings, 13 typos, 14 inappropriate words, and 3 repeated words. 

**Figure 3:**
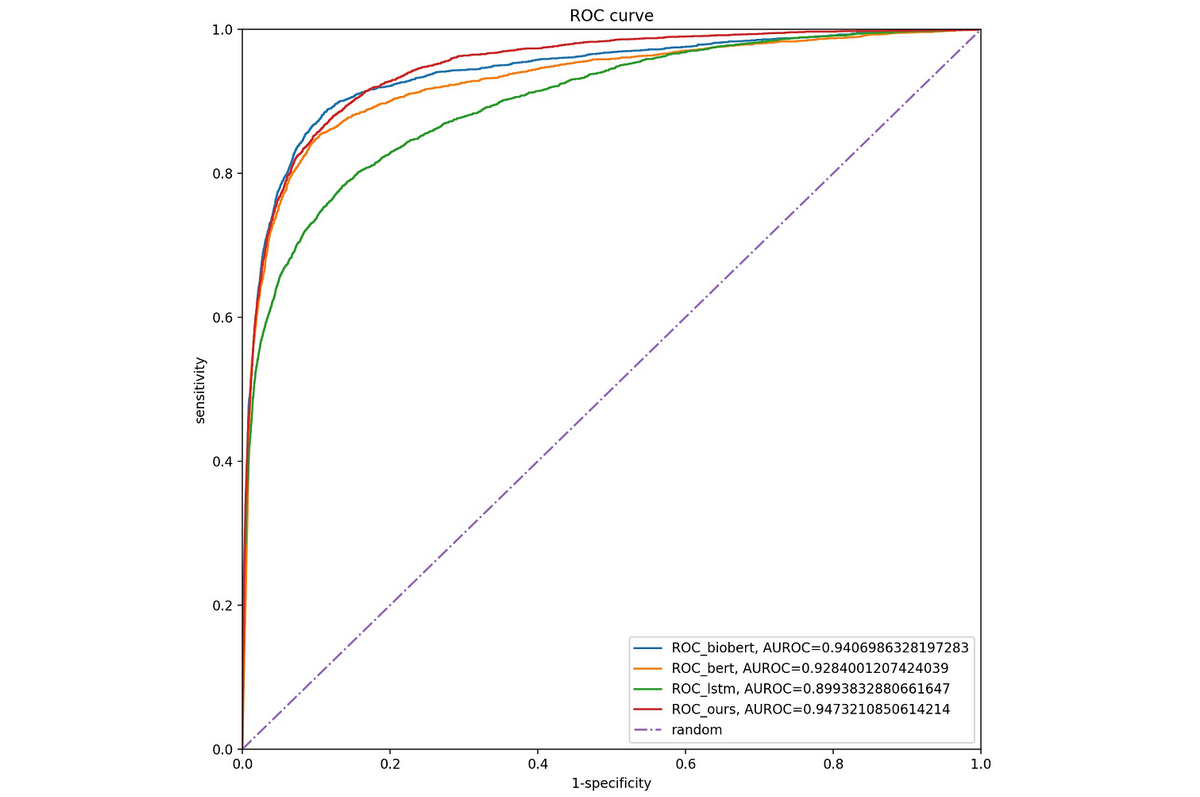
Model receiver operating characteristic (ROC) curves.

Our proposed model, AlphaBERT, demonstrated the highest performance among all compared models with an area under the ROC curve (AUROC) of 0.947, and the LSTM demonstrated the worst performance with an AUROC of 0.899 (Figure 3).

BioBERT achieved the highest ROUGE scores (Table 1). BERT and the proposed model were in the intermediate range, with the lowest scores obtained with the LSTM. In addition, the ROUGE score was the highest for reference Doctor A and was the lowest for Doctor C (Table 1). When there were incorrect words in the input diagnoses, the performance of all models deteriorated (Table 2).

We collected 246 critical scores from the 14 doctors that responded to the questionnaire. Statistically significant differences (based on the paired *t* test) were detected within the LSTM compared to the reference, BERT, BioBERT, and our proposed model, but not with respect to the other models (Table 3).

We built the service on a website [56] using a server with only one CPU (no GPU) on the Microsoft Azure platform to provide a diagnoses-extractive summarization service. Editorial suggestions are also available on the website to gather user feedback and to continue to improve the model. The source code is available on GitHub [57]. The service is currently being integrated into the hospital information system to enhance the capabilities of hospital staff.

**Table 1:** Model parameters and ROUGE^a^ F1 results.

Model		Dr A (n=250)	Dr B (n=248)	Dr C (n=91)		Mean *F1* value	
**BERT^b^ (108,523,714 parameters)**							
	ROUGE-1^c^	0.761	0.693		0.648		0.715
	ROUGE-2^d^	0.612	0.513		0.473		0.549
	ROUGE-L^e^	0.748	0.671		0.627		0.697
**BioBERT^f^ (108,523,714 parameters)**							
	ROUGE-1	0.788	0.697		0.647		0.728
	ROUGE-2	0.642	0.523		0.464		0.565
	ROUGE-L	0.773	0.678		0.629		0.711
**LSTM^g^ (899,841 parameters)**							
	ROUGE-1	0.701	0.647		0.618		0.666
	ROUGE-2	0.531	0.468		0.459		0.494
	ROUGE-L	0.684	0.629		0.602		0.648
**Proposed model (963,496 parameters)**							
	ROUGE-1	0.769	0.678		0.647		0.712
	ROUGE-2	0.610	0.482		0.463		0.533
	ROUGE-L	0.751	0.656		0.632		0.693

^a^ROUGE: Recall-Oriented Understudy for Gisting Evaluation.

^b^BERT: Bidirectional Encoder Representations from Transformers.

^c^ROUGE-1: Recall-Oriented Understudy for Gisting Evaluation with unigram overlap.

^d^ROUGE-2: Recall-Oriented Understudy for Gisting Evaluation with bigram overlap.

^e^ROUGE-L: Recall-Oriented Understudy for Gisting Evaluation for the longest common subsequence (n) representing the number of reference labels.

^f^BioBERT: Bidirectional Encoder Representations from Transformers trained on a biomedical corpus.

^g^LSTM: Long Short-Term Memory.

**Table 2:** ROUGE^a^ F1 results of diagnoses with incorrect words.

ROUGE-L^b^	BERT^c^	BioBERT^d^	LSTM^e^	Proposed Model
Diagnoses without error words (n=451)^f^	0.704	0.717	0.651	0.698
Diagnoses with incorrect words (n=138)	0.676	0.692	0.640	0.674

^a^ROUGE: Recall-Oriented Understudy for Gisting Evaluation.

^b^ROUGE-L: ROUGE for the longest common subsequence.

^c^BERT: Bidirectional Encoder Representations from Transformers.

^d^BioBERT: Bidirectional Encoder Representations from Transformers trained on a biomedical corpus.

^e^LSTM: Long Short-Term Memory.

^f^n represents the number of reference labels.

**Table 3:** Critique scores of models from doctors (N=246).

Model	Score, mean (SD)	*P* value			
		BERT^a^	BioBERT^b^	LSTM^c^	Proposed Model
Reference	2.232 (0.832)	.11	.66	<.001	.10
BERT	2.134 (0.877)		.10	.001	.89
BioBERT	2.207 (0.844)			<.001	.19
LSTM	1.927 (0.910)				.002
Proposed	2.126 (0.874)				

^a^BERT: Bidirectional Encoder Representations from Transformers.

^b^BioBERT: Bidirectional Encoder Representations from Transformers trained on a biomedical corpus.

^c^LSTM: Long Short-Term Memory.

## Discussion

### Principal Findings

AlphaBERT effectively performed the extractive summarization task on medical clinic notes and decreased the model size compared to BERT, reducing the number of parameters from 108,523,714 to 963,496 using a character-level tokenizer. AlphaBERT showed similar performance to BERT and BioBERT in this extractive summarization task. In spite of the heavy model, both BERT and BioBERT were demonstrated to be excellent models and well-suited for several tasks (including the primary task of this study) with small adjustments. For convenience, the model can be used in a straightforward manner to rapidly build new apps in the medical field. Because of the well pretrained NLP feature extraction model, a small label dataset (the fine-tuning training set includes only 2530 cases) is sufficient for supervised learning and achieving the goal. 

In this study, we obtained high ROUGE *F1* scores for all models. In general summarization studies, the ROUGE *F1* score was typically less than 0.40 [6-9], whereas we achieved a score of 0.71, which corresponds with a higher retention ratio (15%) for this task than the corpus of other summarization tasks such as the CNN/Daily Mail Corpus (approximately 7%) [7]. Since the diagnosis can be considered as a summary of admission records, a higher retention rate is reasonable; however, for emergencies, the diagnosis will contain too many redundant words in some cases. 

The ICD-10 is a well-classified system with more than 70,000 codes, but is often too simple to fully capture the complex context of a patient’s record. The treatments during the patient’s previous hospitalization are also important to consider, and are often recorded as a free-text diagnosis when the patient has revisited a hospital under critical status. For example, if a patient has cancer, the previous chemotherapy course is important information when the patient is seriously ill in the emergency department. Furthermore, it is difficult for doctors to accurately find the correct codes; thus, it is insufficient to represent a patient’s condition by simply obtaining the ICD-10 code from the EHR. However, the ICD-10 codes can be used to extend the pretrained training set by random stitching. 

Combining a random number of diagnoses not only extends the training dataset but also improves the performance of the model. The average number of characters in a diagnosis was 355, but the range was larger (SD 318). In the absence of augmentation, the position embeddings and self-attention heads trained more in the front and demonstrated poorer performance in the back. Augmentation combines several diagnoses to lengthen the input embeddings, which can train the self-attention heads to consider all 1350 characters equally.

In the prediction phase, we obtained the probability of each character. Since a word is split into a sequence of characters, the result is fragmented, and only some characters in a word were selected by prediction. This results in a nonsense phrase and produces poor results. Accordingly, we proposed a cleanup method that selects the entire word based on the probability of all characters being present in the word. This concept is derived from the segmentation task in computer vision in which each pixel has the possibility of classifying and causing the predictions to not continue. In the field of computer vision, contour-based superpixels are chosen, and all superpixels are selected by a majority vote [31]. In this study, the average probability of an entire word represents the probability of each character and results in either the entire word being selected or none at all.

Since the summarization task is subjective, properly evaluating the performance of the model is a relevant consideration. Lack of adequate medical labels is an important issue, because labels from qualified physicians are rare and difficult to collect. Although the ROUGE score [54] is widely used in this field, it is evaluated by the same doctors’ labels and even by separate split sets.

Owing to the lack of doctors who are capable of labeling the reference summaries, all of the models evaluated in this study were limited to being fine-tuned by Doctor A’s labels. We were able to shuffle and randomly split the three doctors’ labels to training, validation, and testing sets, but we did not have reference labels from other doctors to confirm whether individual variation exists. Even when using the three doctors’ labels, this problem would occur when gathering another doctor’s labels. 

To confirm the differences from other doctors, the models were fine-tuned using only one doctor’s knowledge, with the others’ used as a test set. The results revealed a difference according to the ROUGE scores (Table 1) from the three doctors. The model had a poor ROUGE score on the label references for Doctor C, implying that summarization is a highly subjective task. Certain words are important for some doctors, but not for others, even among doctors in the same medical field who have similar interpretation processes. Therefore, it was very easy to overfit the model with the summarization task. BioBERT had the most accurate prediction result, but the associated overfitting was also more severe.

We established a website for doctors to easily critique the performance within label references and the predictions from the models to further objectively evaluate the performance of the model and the reference labels from doctors. We used a double-blind method to collect scores, and the system randomly chose a diagnosis and displayed corresponding summary proposals by random ordering. The critical reviewer was therefore blinded to the method used for each prediction. We obtained similar results to the ROUGE scores from this analysis. Moreover, the LSTM was consistently the lowest-performing model, whereas manually labeled references achieved the highest average score, followed by BioBERT.

**Figure 4:**
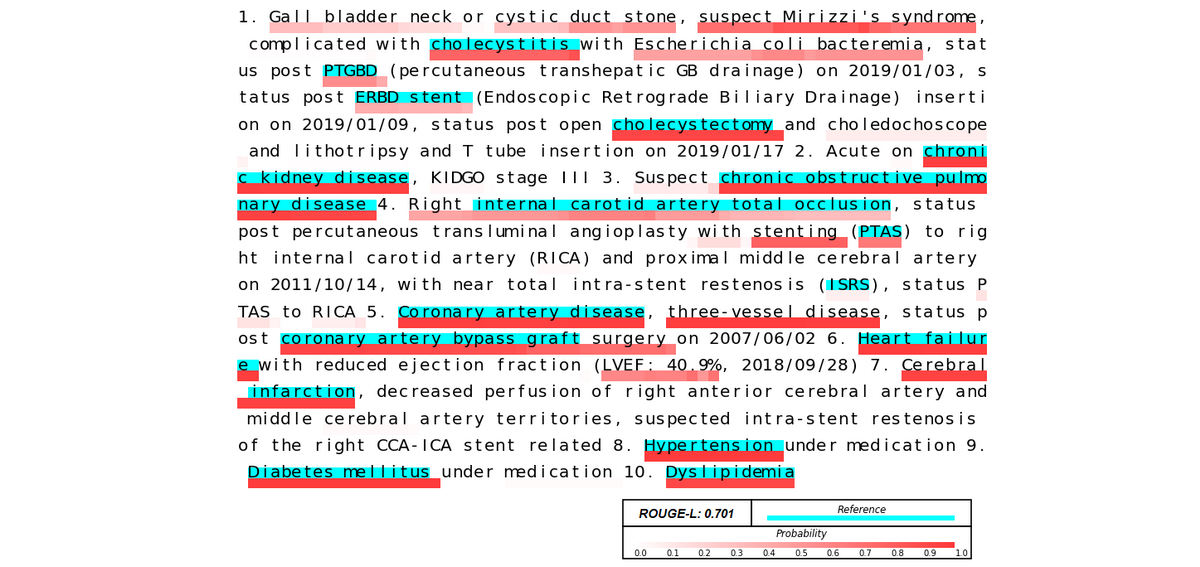
Illustration of the performance of AlphaBERT.

Although the performance of AlphaBERT was not optimum, there was nevertheless no statistically significant difference between the performances of BERT, BioBERT, and AlphaBERT. The advantage of AlphaBERT is the character-level prediction probability and its one-to-one correspondence with the original document. The predicted keywords can be highlighted directly on the original document and can be easily edited by users. For example, although AlphaBERT’s predicted proposal had a ROUGE-L score of 0.701, it makes sense to recognize important words, which is perhaps more informative than a doctor’s reference label (Figure 4). In some cases, our proposed method could predict more information about the disease and related treatments, whereas in other cases some diseases were lost (eg, pneumonia, hypertension, and respiratory failure), and in other cases the formal medical term was predicted but the reference label was an abbreviation (Multimedia Appendix 3). This variation also reflects the subjectivity of the summary task.

### Limitations

Due to the subjective nature of the text summarization task, the predicted summary results may lose some information that may be of relevance. The proposed model helps hospital staff to quickly view information for a large number of patients at the beginning of a shift; however, they will still need to read all of the collected information from the EHRs during ward rounds. 

Typos and misspellings remain a problem in NLP. However, the character-level and word pieces BPE method can not only reduce the vocabulary but can also handle typos effectively to maintain noninferior results (Multimedia Appendix 4). Although automatic spelling correction may be a solution to this problem, we have not included this feature in our proposed method because we are confident in the robust error tolerance of the character-level and BPE method.

This was a pilot study in the medical text summarization field based on the deep-learning method. We plan to establish a website that offers this service and provides a way to edit suggestions and feedback to collect volunteer labels and resolve personal variability in the near future. 

### Conclusions

AlphaBERT, using character-level tokens in a BERT-based model, can greatly decrease model size without significantly reducing performance for text summarization tasks. The proposed model will provide a method to further extract the unstructured free-text portions in EHRs to obtain an abundance of health data. As we enter the forefront of the artificial intelligence era, NLP deep-learning models are well under development. In our model, all medical free-text data can be transformed into meaningful embeddings, which will enhance medical studies and strengthen doctors’ capabilities.

## Appendix

Multimedia Appendix 1 Input embedding.

Multimedia Appendix 2 Flowchart to determine the hyperparameters and measure the model’s performance.

Multimedia Appendix 3 Error statistics (strong and weak).

Multimedia Appendix 4 Error statistics (typos, misspellings, or incorrect words).

## Abbreviations

AUROCs: Area Under the Receiver Operating Characteristics
BERT: Bidirectional Encoder Representations from Transformers
BPE: byte-pair encoding
EHR: electronic health record
ICD-10: International Statistical Classification of Diseases and Related Health Problems 10th Revision
LSTM: long short-term memory
NLP: natural language processing
NTUH-iMD: National Taiwan University Hospital Integrated Medical Database
ROC: receiver operating characteristic
ROUGE: Recall-Oriented Understudy for Gisting Evaluation
