# Supplementary figures and images for: Modified Bidirectional Encoder Representations From Transformers Extractive Summarization Model for Hospital Information Systems Based on Character-Level Tokens (AlphaBERT): Development and Performance Evaluation
Source: JMIR Med Inform. 2020 Apr 29;8(4):e17787. doi: 10.2196/17787 (PMC7221648; doi:10.2196/17787)

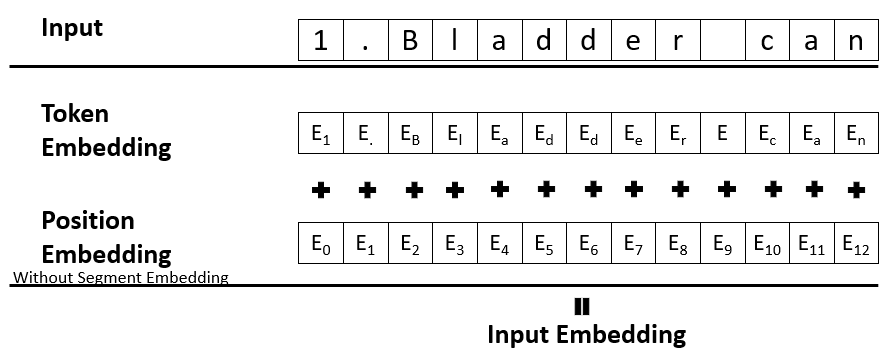

Supplement: Multimedia Appendix 1 [file medinform_v8i4e17787_app1.PNG]

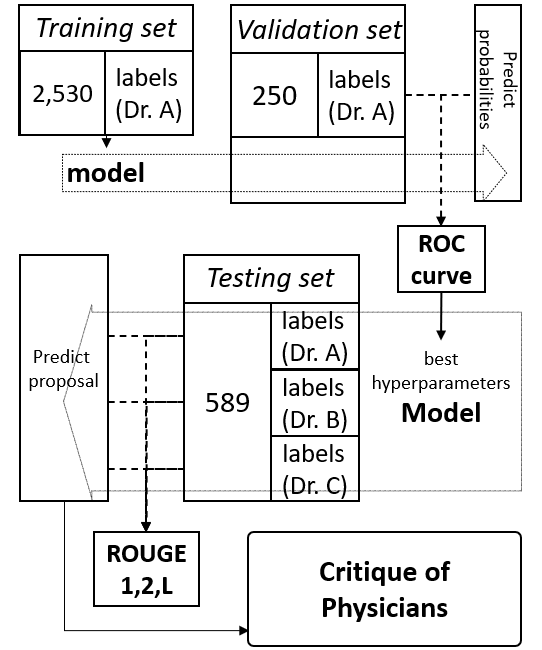

Supplement: Multimedia Appendix 2 [file medinform_v8i4e17787_app2.PNG]
