# Supplementary material for: Modified Bidirectional Encoder Representations From Transformers Extractive Summarization Model for Hospital Information Systems Based on Character-Level Tokens (AlphaBERT): Development and Performance Evaluation
Source: JMIR Med Inform. 2020 Apr 29;8(4):e17787. doi: 10.2196/17787 (PMC7221648; doi:10.2196/17787)
Supplement: Multimedia Appendix 3 [file medinform_v8i4e17787_app3.pdf]

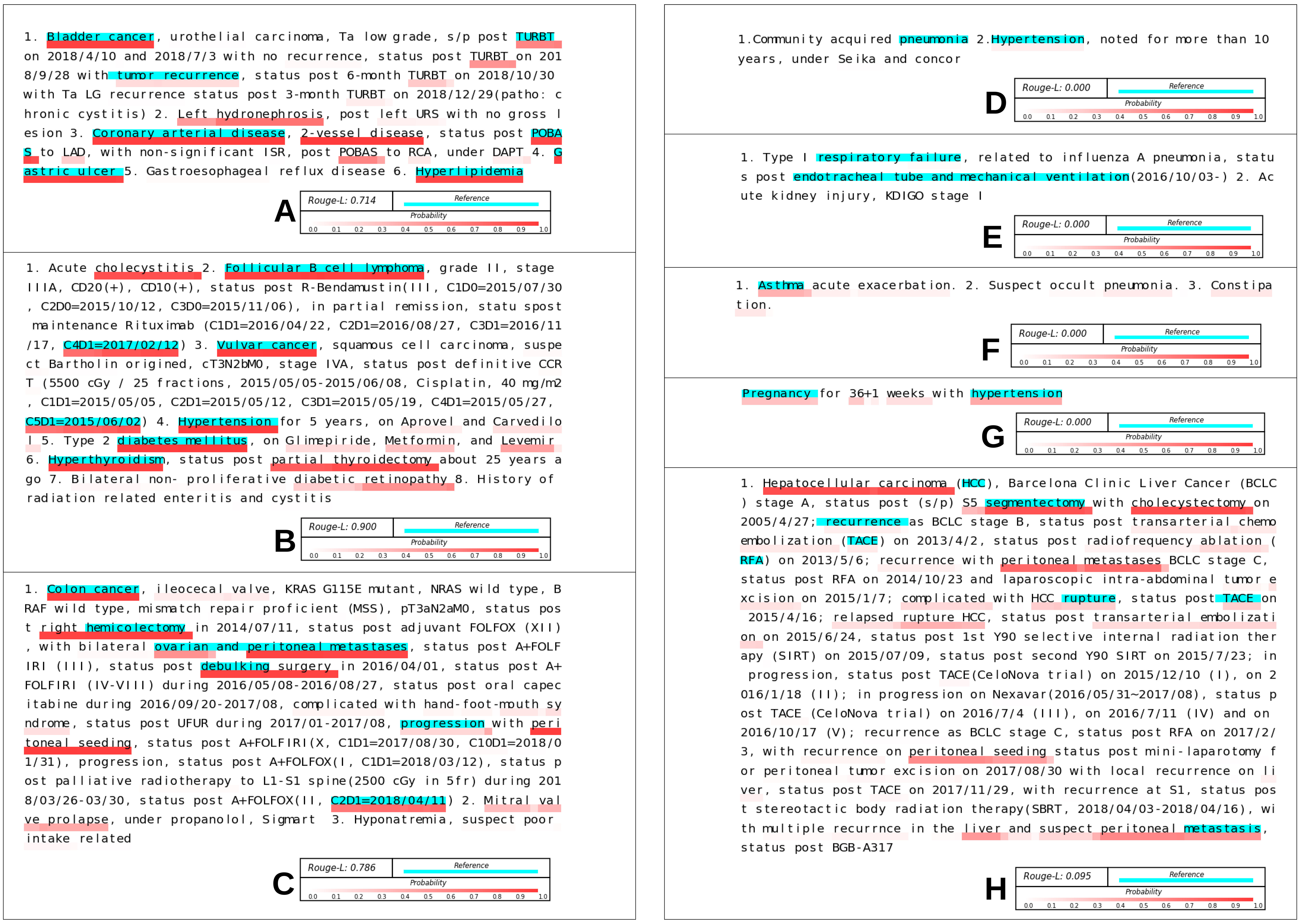
1. Bladder cancer, urothelial carcinoma, Ta low grade, s/p post TURBT
on 2018/4/10 and 2018/7/3 with no recurrence, status post TURBT on 201
8/9/28 with tumor recurrence, status post 6-month TURBT on 2018/10/30
with Ta LG recurrence status post 3-month TURBT on 2018/12/29(patho: c
hronic cystitis) 2. Left hydronephrosis, post left URS with no gross l
esion 3. Coronary arterial disease, 2-vessel disease, status post POBA
S to LAD, with non-significant ISR, post POBAS to RCA, under DAPT 4. G
astric ulcer 5. Gastroesophageal reflux disease 6. Hyperlipidemia
A
Rouge-L: 0.714
Reference
Probability
0.0 0.1 0.2 0.3 0.4 0.5 0.6 0.7 0.8 0.9 1.0
1. Acute cholecystitis 2. Follicular B cell lymphoma, grade II, stage
IIIA, CD20(+), CD10(+), status post R-Bendamustin(III, C1D0=2015/07/30
, C2D0=2015/10/12, C3D0=2015/11/06), in partial remission, statu spost
maintenance Rituximab (C1D1=2016/04/22, C2D1=2016/08/27, C3D1=2016/11
/17, C4D1=2017/02/12) 3. Vulvar cancer, squamous cell carcinoma, suspe
ct Bartholin origined, cT3N2bM0, stage IVA, status post definitive CCR
T (5500 cGy / 25 fractions, 2015/05/05-2015/06/08, Cisplatin, 40 mg/m2
, C1D1=2015/05/05, C2D1=2015/05/12, C3D1=2015/05/19, C4D1=2015/05/27,
C5D1=2015/06/02) 4. Hypertension for 5 years, on Aprovel and Carvedilo
l 5. Type 2 diabetes mellitus, on Glimepiride, Metformin, and Levemir
6. Hyperthyroidism, status post partial thyroidectomy about 25 years a
go 7. Bilateral non- proliferative diabetic retinopathy 8. History of
radiation related enteritis and cystitis
B
Rouge-L: 0.900
Reference
Probability
0.0 0.1 0.2 0.3 0.4 0.5 0.6 0.7 0.8 0.9 1.0
1. Colon cancer, ileocecal valve, KRAS G115E mutant, NRAS wild type, B
RAF wild type, mismatch repair proficient (MSS), pT3aN2aM0, status pos
t right hemicolectomy in 2014/07/11, status post adjuvant FOLFOX (XII)
, with bilateral ovarian and peritoneal metastases, status post A+FOLF
IRI (III), status post debulking surgery in 2016/04/01, status post A+
FOLFIRI (IV-VIII) during 2016/05/08-2016/08/27, status post oral capec
itabine during 2016/09/20-2017/08, complicated with hand-foot-mouth sy
ndrome, status post UFUR during 2017/01-2017/08, progression with peri
toneal seeding, status post A+FOLFIRI(X, C1D1=2017/08/30, C10D1=2018/0
1/31), progression, status post A+FOLFOX(I, C1D1=2018/03/12), status p
ost palliative radiotherapy to L1-S1 spine(2500 cGy in 5fr) during 201
8/03/26-03/30, status post A+FOLFOX(II, C2D1=2018/04/11) 2. Mitral val
ve prolapse, under propanolol, Sigmart 3. Hyponatremia, suspect poor
intake related
C
Rouge-L: 0.786
Reference
Probability
0.0 0.1 0.2 0.3 0.4 0.5 0.6 0.7 0.8 0.9 1.0
1.Community acquired pneumonia 2.Hypertension, noted for more than 10
years, under Seika and concor
D
Rouge-L: 0.000
Reference
Probability
0.0 0.1 0.2 0.3 0.4 0.5 0.6 0.7 0.8 0.9 1.0
1. Type I respiratory failure, related to influenza A pneumonia, statu
s post endotracheal tube and mechanical ventilation(2016/10/03-) 2. Ac
ute kidney injury, KDIGO stage I
E
Rouge-L: 0.000
Reference
Probability
0.0 0.1 0.2 0.3 0.4 0.5 0.6 0.7 0.8 0.9 1.0
1. Asthma acute exacerbation. 2. Suspect occult pneumonia. 3. Constipa
tion.
F
Rouge-L: 0.000
Reference
Probability
0.0 0.1 0.2 0.3 0.4 0.5 0.6 0.7 0.8 0.9 1.0
Pregnancy for 36+1 weeks with hypertension
G
Rouge-L: 0.000
Reference
Probability
0.0 0.1 0.2 0.3 0.4 0.5 0.6 0.7 0.8 0.9 1.0
1. Hepatocellular carcinoma (HCC), Barcelona Clinic Liver Cancer (BCLC
) stage A, status post (s/p) S5 segmentectomy with cholecystectomy on
2005/4/27; recurrence as BCLC stage B, status post transarterial chemo
embolization (TACE) on 2013/4/2, status post radiofrequency ablation (
RFA) on 2013/5/6; recurrence with peritoneal metastases BCLC stage C,
status post RFA on 2014/10/23 and laparoscopic intra-abdominal tumor e
xcision on 2015/1/7; complicated with HCC rupture, status post TACE on
2015/4/16; relapsed rupture HCC, status post transarterial embolizati
on on 2015/6/24, status post 1st Y90 selective internal radiation ther
apy (SIRT) on 2015/07/09, status post second Y90 SIRT on 2015/7/23; in
progression, status post TACE(CeloNova trial) on 2015/12/10 (I), on 2
016/1/18 (II); in progression on Nexavar(2016/05/31~2017/08), status p
ost TACE (CeloNova trial) on 2016/7/4 (III), on 2016/7/11 (IV) and on
2016/10/17 (V); recurrence as BCLC stage C, status post RFA on 2017/2/
3, with recurrence on peritoneal seeding status post mini-laparotomy f
or peritoneal tumor excision on 2017/08/30 with local recurrence on li
ver, status post TACE on 2017/11/29, with recurrence at S1, status pos
t stereotactic body radiation therapy(SBRT, 2018/04/03-2018/04/16), wi
th multiple recurrnce in the liver and suspect peritoneal metastasis,
status post BGB-A317
H
Rouge-L: 0.095
Reference
Probability
0.0 0.1 0.2 0.3 0.4 0.5 0.6 0.7 0.8 0.9 1.0
