# Supplementary material for: Modified Bidirectional Encoder Representations From Transformers Extractive Summarization Model for Hospital Information Systems Based on Character-Level Tokens (AlphaBERT): Development and Performance Evaluation
Source: JMIR Med Inform. 2020 Apr 29;8(4):e17787. doi: 10.2196/17787 (PMC7221648; doi:10.2196/17787)
Supplement: Multimedia Appendix 4 [file medinform_v8i4e17787_app4.pdf]

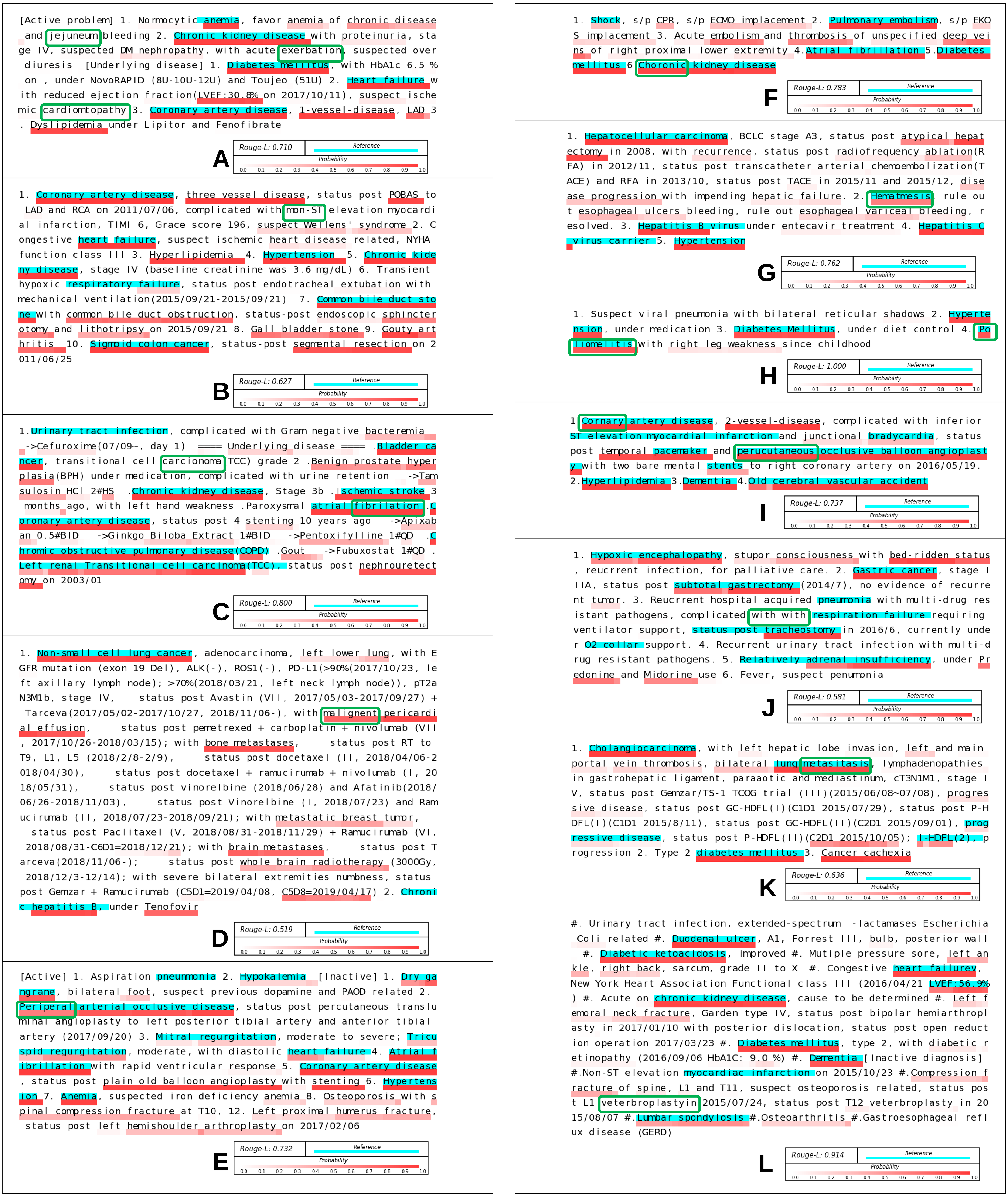
[Active problem] 1. Normocytic anemia, favor anemia of chronic disease and jejuneum bleeding 2. Chronic kidney disease with proteinuria, stage IV, suspected DM nephropathy, with acute exerbation, suspected over diuresis [Underlying disease] 1. Diabetes mellitus, with HbA1c 6.5 % on , under NovoRAPID (8U-10U-12U) and Toujeo (51U) 2. Heart failure with reduced ejection fraction(LVEF:30.8% on 2017/10/11), suspect ischemic cardiomtopathy 3. Coronary artery disease, 1-vessel-disease, LAD 3. Dyslipidemia under Lipitor and Fenofibrate
A
Rouge-L: 0.710
Reference
Probability
0.0 0.1 0.2 0.3 0.4 0.5 0.6 0.7 0.8 0.9 1.0
1. Coronary artery disease, three vessel disease, status post POBAS to LAD and RCA on 2011/07/06, complicated with mon-ST elevation myocardial infarction, TIMI 6, Grace score 196, suspect Wellens' syndrome 2. Congestive heart failure, suspect ischemic heart disease related, NYHA function class III 3. Hyperlipidemia 4. Hypertension 5. Chronic kidney disease, stage IV (baseline creatinine was 3.6 mg/dL) 6. Transient hypoxic respiratory failure, status post endotracheal extubation with mechanical ventilation(2015/09/21-2015/09/21) 7. Common bile duct stone with common bile duct obstruction, status-post endoscopic sphincterotomy and lithotripsy on 2015/09/21 8. Gall bladder stone 9. Gouty arthritis 10. Sigmoid colon cancer, status-post segmental resection on 2011/06/25
B
Rouge-L: 0.627
Reference
Probability
0.0 0.1 0.2 0.3 0.4 0.5 0.6 0.7 0.8 0.9 1.0
1.Urinary tract infection, complicated with Gram negative bacteremia ->Cefuroxime(07/09~, day 1) ==== Underlying disease ==== .Bladder cancer, transitional cell carcionoma(TCC) grade 2 .Benign prostate hyperplasia(BPH) under medication, complicated with urine retention ->Tamsulosin HCl 2#HS .Chronic kidney disease, Stage 3b .Ischemic stroke 3 months ago, with left hand weakness .Paroxysmal atrial fibrillation .Coronary artery disease, status post 4 stenting 10 years ago ->Apixaban 0.5#BID ->Ginkgo Biloba Extract 1#BID ->Pentoxifylline 1#QD .Chronic obstructive pulmonary disease(COPD) .Gout ->Fubuxostat 1#QD .Left renal Transitional cell carcinoma(TCC), status post nephroureterectomy on 2003/01
C
Rouge-L: 0.800
Reference
Probability
0.0 0.1 0.2 0.3 0.4 0.5 0.6 0.7 0.8 0.9 1.0
1. Non-small cell lung cancer, adenocarcinoma, left lower lung, with EGFR mutation (exon 19 Del), ALK(-), ROS1(-), PD-L1(>90%(2017/10/23, left axillary lymph node); >70%(2018/03/21, left neck lymph node)), pT2aN3M1b, stage IV, status post Avastin (VII, 2017/05/03-2017/09/27) + Tarceva(2017/05/02-2017/10/27, 2018/11/06-), with malignent pericardial effusion, status post pemetrexed + carboplatin + nivolumab (VII, 2017/10/26-2018/03/15); with bone metastases, status post RT to T9, L1, L5 (2018/2/8-2/9), status post docetaxel (II, 2018/04/06-2018/04/30), status post docetaxel + ramucirumab + nivolumab (I, 2018/05/31), status post vinorelbine (2018/06/28) and Afatinib(2018/06/26-2018/11/03), status post Vinorelbine (I, 2018/07/23) and Ramucirumab (II, 2018/07/23-2018/09/21); with metastatic breast tumor, status post Paclitaxel (V, 2018/08/31-2018/11/29) + Ramucirumab (VI, 2018/08/31-C6D1=2018/12/21); with brain metastases, status post Tarceva(2018/11/06-); status post whole brain radiotherapy (3000Gy, 2018/12/3-12/14); with severe bilateral extremities numbness, status post Gemzar + Ramucirumab (C5D1=2019/04/08, C5D8=2019/04/17) 2. Chronic hepatitis B, under Tenofovir
D
Rouge-L: 0.519
Reference
Probability
0.0 0.1 0.2 0.3 0.4 0.5 0.6 0.7 0.8 0.9 1.0
[Active] 1. Aspiration pneummonia 2. Hypokalemia [Inactive] 1. Dry gangrane, bilateral foot, suspect previous dopamine and PAOD related 2. Periperal arterial occlusive disease, status post percutaneous transluminal angioplasty to left posterior tibial artery and anterior tibial artery (2017/09/20) 3. Mitral regurgitation, moderate to severe; Tricuspid regurgitation, moderate, with diastolic heart failure 4. Atrial fibrillation with rapid ventricular response 5. Coronary artery disease, status post plain old balloon angioplasty with stenting 6. Hypertension 7. Anemia, suspected iron deficiency anemia 8. Osteoporosis with spinal compression fracture at T10, 12. Left proximal humerus fracture, status post left hemishoulder arthroplasty on 2017/02/06
E
Rouge-L: 0.732
Reference
Probability
0.0 0.1 0.2 0.3 0.4 0.5 0.6 0.7 0.8 0.9 1.0
1. Shock, s/p CPR, s/p ECMO implacement 2. Pulmonary embolism, s/p EKOS implacement 3. Acute embolism and thrombosis of unspecified deep veins of right proximal lower extremity 4.Atrial fibrillation 5.Diabetes mellitus 6 Choronic kidney disease
F
Rouge-L: 0.783
Reference
Probability
0.0 0.1 0.2 0.3 0.4 0.5 0.6 0.7 0.8 0.9 1.0
1. Hepatocellular carcinoma, BCLC stage A3, status post atypical hepatectomy in 2008, with recurrence, status post radiofrequency ablation(RFA) in 2012/11, status post transcatheter arterial chemoembolization(TACE) and RFA in 2013/10, status post TACE in 2015/11 and 2015/12, disease progression with impending hepatic failure. 2. Hematmesis, rule out esophageal ulcers bleeding, rule out esophageal variceal bleeding, resolved. 3. Hepatitis B virus under entecavir treatment 4. Hepatitis C virus carrier 5. Hypertension
G
Rouge-L: 0.762
Reference
Probability
0.0 0.1 0.2 0.3 0.4 0.5 0.6 0.7 0.8 0.9 1.0
1. Suspect viral pneumonia with bilateral reticular shadows 2. Hypertension, under medication 3. Diabetes Mellitus, under diet control 4. Poliomelitis with right leg weakness since childhood
H
Rouge-L: 1.000
Reference
Probability
0.0 0.1 0.2 0.3 0.4 0.5 0.6 0.7 0.8 0.9 1.0
1.Cornary artery disease, 2-vessel-disease, complicated with inferior ST elevation myocardial infarction and junctional bradycardia, status post temporal pacemaker and perucutaneous occlusive balloon angioplasty with two bare mental stents to right coronary artery on 2016/05/19. 2.Hyperlipidemia 3.Dementia 4.Old cerebral vascular accident
I
Rouge-L: 0.737
Reference
Probability
0.0 0.1 0.2 0.3 0.4 0.5 0.6 0.7 0.8 0.9 1.0
1. Hypoxic encephalopathy, stupor consciousness with bed-ridden status, reucrrent infection, for palliative care. 2. Gastric cancer, stage IIIA, status post subtotal gastrectomy (2014/7), no evidence of recurrent tumor. 3. Reucrrent hospital acquired pneumonia with multi-drug resistant pathogens, complicated with with respiration failure requiring ventilator support, status post tracheostomy in 2016/6, currently under O2 collar support. 4. Recurrent urinary tract infection with multi-drug resistant pathogens. 5. Relatively adrenal insufficiency, under Predonine and Midorine use 6. Fever, suspect penumonia
J
Rouge-L: 0.581
Reference
Probability
0.0 0.1 0.2 0.3 0.4 0.5 0.6 0.7 0.8 0.9 1.0
1. Cholangiocarcinoma, with left hepatic lobe invasion, left and main portal vein thrombosis, bilateral lung metasitasis, lymphadenopathies in gastrohepatic ligament, paraaotic and mediastinum, cT3N1M1, stage IV, status post Gemzar/TS-1 TCOG trial (III)(2015/06/08~07/08), progressive disease, status post GC-HDFL(I)(C1D1 2015/07/29), status post P-HDFL(I)(C1D1 2015/8/11), status post GC-HDFL(II)(C2D1 2015/09/01), progressive disease, status post P-HDFL(II)(C2D1 2015/10/05); I-HDFL(2), progression 2. Type 2 diabetes mellitus 3. Cancer cachexia
K
Rouge-L: 0.636
Reference
Probability
0.0 0.1 0.2 0.3 0.4 0.5 0.6 0.7 0.8 0.9 1.0
#. Urinary tract infection, extended-spectrum -lactamases Escherichia Coli related #. Duodenal ulcer, A1, Forrest III, bulb, posterior wall #. Diabetic ketoacidosis, improved #. Mutiple pressure sore, left ankle, right back, sarcum, grade II to X #. Congestive heart failurev, New York Heart Association Functional class III (2016/04/21 LVEF:56.9%) #. Acute on chronic kidney disease, cause to be determined #. Left femoral neck fracture, Garden type IV, status post bipolar hemiarthroplasty in 2017/01/10 with posterior dislocation, status post open reduction operation 2017/03/23 #. Diabetes mellitus, type 2, with diabetic retinopathy (2016/09/06 HbA1C: 9.0 %) #. Dementia [Inactive diagnosis] #.Non-ST elevation myocardiac infarction on 2015/10/23 #.Compression fracture of spine, L1 and T11, suspect osteoporosis related, status post L1 veterbroplastyin 2015/07/24, status post T12 veterbroplasty in 2015/08/07 #.Lumbar spondylosis #.Osteoarthritis #.Gastroesophageal reflux disease (GERD)
L
Rouge-L: 0.914
Reference
Probability
0.0 0.1 0.2 0.3 0.4 0.5 0.6 0.7 0.8 0.9 1.0
